# Reduced immune cell infiltration and increased pro-inflammatory mediators in the brain of Type 2 diabetic mouse model infected with West Nile virus

**DOI:** 10.1186/1742-2094-11-80

**Published:** 2014-04-21

**Authors:** Mukesh Kumar, Kelsey Roe, Pratibha V Nerurkar, Beverly Orillo, Karen S Thompson, Saguna Verma, Vivek R Nerurkar

**Affiliations:** 1 Department of Tropical Medicine, Medical Microbiology and Pharmacology, John A. Burns School of Medicine, University of Hawaii at Manoa, 651 Ilalo Street, BSB 320G, Honolulu, Hawaii 96813, USA; 2 Pacific Center for Emerging Infectious Diseases Research, John A. Burns School of Medicine, University of Hawaii at Manoa, 651 Ilalo Street, BSB 320G, Honolulu, Hawaii 96813, USA; 3 Department of Molecular Biosciences and Bioengineering, Laboratory of Metabolic Disorders and Alternative Medicine, College of Tropical Agriculture and Human Resources, University of Hawaii at Manoa, 1955 East-West Road, Honolulu, Hawaii 96822, USA; 4 Department of Pathology, John A. Burns School of Medicine, University of Hawaii at Manoa, 651 Ilalo Street, BSB 320G, Honolulu, Hawaii 96813, USA

**Keywords:** West Nile virus encephalitis, Type 2 diabetes, Neuroinflammation, Cell adhesion molecules, CD8^+^ T cells, Neurons

## Abstract

**Background:**

Diabetes is a significant risk factor for developing West Nile virus (WNV)-associated encephalitis (WNVE) in humans, the leading cause of arboviral encephalitis in the United States. Using a diabetic mouse model (*db/db*), we recently demonstrated that diabetes enhanced WNV replication and the susceptibility of mice to WNVE. Herein, we have examined immunological events in the brain of wild type (WT) and *db/db* mice after WNV infection. We hypothesized that WNV-induced migration of protective leukocytes into the brain is attenuated in the presence of diabetes, leading to a high viral load in the brain and severe disease in diabetic mice.

**Methods:**

Nine-week old C57BL/6 WT and *db/db* mice were infected with WNV. Leukocyte infiltration, expression of cell adhesion molecules (CAM), neuroinflammatory responses, activation of astrocytes, and neuronal death were analyzed using immunohistochemistry, qRT-PCR, flow cytometry, and western blot.

**Results:**

We demonstrate that infiltration of CD45^+^ leukocytes and CD8^+^T cells was significantly reduced in the brains of *db/db* mice, which was correlated with attenuated expression of CAM such as E-selectin and ICAM-1. WNV infection in *db/db* mice was associated with an enhanced inflammatory response in the brain. mRNA and protein levels of key chemokines such as CXCL10, CXCL1, CCL2, CCL5, CCL3, and G-CSF, and cytokines such as IL-1β, TNF, IL-6, IFNγ, and IL-1α were significantly elevated in the brains of *db/db* mice compared to WT mice. Elevated levels of cytokines also correlated with increased astrocytes activation and neuronal damage in the brains of *db/db* mice.

**Conclusion:**

These data suggest that reduced leukocytes recruitment, in part, due to lower levels of CAM results in failure to clear WNV infection from the brain leading to increased production of inflammatory molecules, which mediates increased neuronal death and mortality in *db/db* mice. This is the first study to elucidate the expression of CAM and their correlation with the migration of leukocytes, specifically cytotoxic CD8^+^ T cells, in increasing disease severity in the diabetic mouse model.

## Background

Type 2 diabetes is associated with an impaired immune response and increased susceptibility to various pathogens [1,2]. Diabetes inhibits important aspects of leukocyte function including adhesion, chemotaxis and phagocytosis, oxidative burst, and intracellular killing [2-5]. Diabetes is also associated with an enhanced inflammatory response to infections [6,7]. It is known that diabetes adversely affects leukocyte adherence and transendothelial migration. Several studies have demonstrated that the adhesion and migration of neutrophils and monocytes in response to various chemotactic stimuli is significantly reduced in diabetic patients compared to non-diabetic controls [3,8,9]. It has also been demonstrated that diabetes results in attenuated upregulation of cell adhesion molecules (CAM) and their ligands, such as intercellular cell adhesion molecule 1 (ICAM-1), E-selectin, and macrophage antigen-1 after various stimuli such as lipopolysaccharides (LPS) [3,10,11]. However the effect of diabetes on the expression of CAM and the migration of leukocytes, specifically across the blood-brain barrier (BBB), in response to viral infections such as West Nile virus (WNV) remains largely unknown. Moreover, the effect of diabetes on the migration of cytotoxic CD8^+^ T cells, important cells in protection against viral infections, has never been studied.

WNV, a mosquito-borne *Flavivirus,* belonging to the family *Flaviviridae*, that causes lethal encephalitis, has emerged as a significant cause of viral encephalitis in the United States [12]. Since its introduction to North America in 1999, outbreaks of WNV fever (WNF) and encephalitis (WNVE) have occurred in regions throughout United States. Up to 70% of the survivors of WNV neuroinvasive disease experience persistent neurological deficits after infection [13]. There are no therapeutic agents or vaccines approved for use against WNV infection in humans. WNVE occurs more frequently in elderly and in persons with a compromised immune system, hypertension and Type 2 diabetes [14]. Epidemiological and experimental data suggests that diabetes is associated with an increased risk of WNVE [15-19], and that patients with diabetes are four times more likely to develop WNVE than WNF, which is significantly more than other factors such as old age, male gender and hypertension [20,21]. It has been reported that a higher proportion of patients with WNV infection had hyperglycemia on admission to the hospital. Moreover, persons with diabetes are also most likely to have persistent symptoms after WNV infection [22,23]. Despite its public health importance, the neuropathogenesis of WNV infection in the diabetic population is largely unknown.

WNVE is characterized by neuronal death, activation of glial cells, and infiltration of leukocytes in the perivascular space and parenchyma [24-26]. A robust induction of antiviral immune responses is critical for the control of WNV infection in the periphery and brain. Antiviral type I interferon (IFN-α and -β) production is essential in suppressing viral titers in the brain and peripheral organs [27]. The induction of WNV-specific immunoglobulins (IgM and IgG) is essential for suppressing viremia and virus dissemination [28]. The migration of leukocytes into the brain is essential for controlling WNV infection in the brain [25,29]. CD8^+^ T cells are essential for protection against WNV infection by clearing the virus in the brain [30]. An absence of CD8^+^ T cells results in unrestricted WNV replication and increased neuronal death because of the high cytopathic potential of WNV [24,30]. WNV-induced expression of CAM, such as ICAM-1, and pro-inflammatory molecules such as tumor necrosis factor (TNF) and CXCL10 promote the trafficking of leukocytes into the brain [29,31,32].

We have previously demonstrated, using the diabetic mouse model *db/db*, that diabetes enhanced the susceptibility of mice to WNV disease and suppressed virus clearance in serum, peripheral tissues, and brains of *db/db* mice when compared to wild type (WT) mice [19]. These observations were associated with a significant delay in the induction of antiviral immune responses (IFN-α, IgM, and IgG) and an increase in the pro-inflammatory responses in the serum of *db/db* mice. In this study, we have analyzed the immunological events in the brains of WT and *db/db* mice after WNV infection in order to understand the immune mechanisms underlying increased WNV disease severity in diabetics.

## Methods

### Animal experiments

Male nine-week-old C57BL/6 J-*Lepr*^*db*^*/Lepr*^*db*^*(db/db)* mice and C57BL/6 J (WT) mice were purchased from The Jackson Laboratory (Bar Harbor, Maine, United States). Animals were housed four per cage and allowed to eat and drink freely. The animal suite was maintained at 72°F, at 45% humidity, and on 12 hour light and dark cycles. Sawdust bedding was provided along with paper towels. Trained and certified personnel conducted all the animal experiments. This study was approved by the University of Hawaii Institutional Animal Care and Use Committee (IACUC) (protocol number 10-948), and was conducted in strict accordance with guidelines established by the National Institutes of Health and the University of Hawaii IACUC.

After acclimatization for one week, WT and *db/db* mice were inoculated via the footpad route with 10 plaque forming units (PFU) of WNV (NY99) or phosphate buffered saline (PBS, mock) and at days 4, 6, and 8 after infection. Mice were anesthetized using isoflurane and perfused with cold PBS as described previously [19,33,34]. Brains were harvested and flash frozen in 2-methylbutane (Sigma, St. Louis, Missouri, United States) and stored at -80°C until further processing. WNV is first detected in the brain between days 4 and 6 after footpad inoculation and peak virus load is observed at day 8 after infection [34], therefore, brains were harvested at days 4, 6, and 8 after infection.

Alternatively, mice were perfused with PBS followed by 4% paraformaldehyde (PFA) and brains were harvested, cryoprotected in 30% sucrose (Sigma, St. Louis, Missouri, United States), and embedded in the optimum cutting temperature (OCT) as described previously [34]. One half of the frozen brain tissues were weighed and homogenized in a bullet blender using glass or zirconium beads, and a plaque assay was conducted as described previously [19].

### Quantitation by qRT-PCR and western blot

One half of the frozen brain tissues were powdered over dry ice to obtain a homogenous sampling and an aliquot of the frozen brain powder was used to extract total RNA and protein. The mRNA levels of multiple host genes were determined using qRT-PCR and the fold-change in infected brains compared to mock brains was calculated after normalizing to the *β-actin* gene as described previously [34-36]. The primer sequences and annealing temperatures used for qRT-PCR are listed in Table 1. Total cellular protein was extracted from the brain and 20 to 30 μg protein was separated on SDS-PAGE, transferred onto nitrocellulose membrane and incubated overnight with polyclonal antibodies against ICAM-1, E-selectin, vascular cell adhesion molecule 1 (VCAM-1) (Santa Cruz, Dallas, Texas, United States) and β-actin (Sigma, St. Louis, Missouri, United States) as described previously [34,35,37]. Following incubation with secondary antibodies conjugated with IRDye 800 and IRDye 680 (Li-Cor Biosciences, Lincoln, Nebraska United States), the membranes were scanned using the Odyssey infrared imager (Li-Cor Biosciences, Lincoln, Nebraska United States).

**Table 1:** **Primer sequences used for qRT**-**PCR**

**Gene (Accession No.)**	**Primer Sequence (5′-3′)**	**Amplicon**	
		(bp)*	**Tm (°C)**^**#**^
*CD45* [GenBank: L36091]			
Forward	GCCCAAACAAATTACACAT	107	58
Reverse	TTAGGCGTTTCTGGAATC		
*CD4* [GenBank: NM_013488]			
Forward	GGAAGACTCTCAGACTTAT	79	57
Reverse	GAAGGTCACTTTGAACAC		
*CD8* [GenBank: NM_009857]			
Forward	GTAATGAGCAGACTGTAAC	79	56
Reverse	CTATATGATGGGCAGACA		
*CD11b* [GenBank: EF101557]			
Forward	GCATGTCAAGAACAAGTA	133	56
Reverse	CTAAAGCCAGGTCATAAG		
*GFAP* [GenBank: NM_010277]			
Forward	GTGGATTTGGAGAGAAAG	177	56
Reverse	GTATTGAGTGCGAATCTC		
*ICAM-1* [GenBank: NM_010493]			
Forward	ATAACTGGACTATAATCATTCTG	119	57
Reverse	AGCCTTCTGTAACTTGTAT		
*E-selectin* [GenBank: M87862]			
Forward	CATGACGTATGATGAAGC	98	57
Reverse	GATTGGAGTTAAGGTAGTTG		
*VCAM-1* [GenBank: NM_011693]			
Forward	CTCTAGCAAGACCCTTTA	149	57
Reverse	CATCTTCACAGGCATTTC		

*bp, base pair; ^**#**^*Tm*, Melting temperature.

### Immunohistochemistry

Sagittal sections of 10-μm thickness were cut from the hemi-brain tissues frozen in OCT, and whole sagittal tissue sections were stained with anti-CD45-FITC and anti-CD8-APC (eBiosciences, San Diego, California, United States) overnight at 4°C as described previously [34,37]. Additionally, tissue sections were also incubated with primary antibodies against glial fibrillary acidic protein (GFAP) (DakoCytomation, Carpinteria, California, United States), NeuN (Millipore, Billerica, Massachusetts, United States), E-selectin, ICAM-1, VCAM-1 (Santa Cruz, Dallas, Texas, United States) and von Willebrand factor (vWF) (Abcam, Cambridge, Massachusetts, United States) overnight at 4°C followed by Alexa Fluor 546 or Alexa Fluor 488 conjugated secondary antibody as described previously [34]. Terminal deoxynucleotidyl transferase dUTP nick end labeling (TUNEL) staining was conducted using an *in situ* cell death detection kit (Roche, Indianapolis, Indiana, United States) as described previously [38]. Images were acquired using the Zeiss Axiovert 200 fluorescent microscope (Carl Zeiss Microscopy, Thornwood, New York, United States).

### Flow cytometric analysis of infiltrated immune cells in the brain and spleen

The brains and spleens from three WT and *db/db* mice (two independent experiments, a total of six mice per group) were isolated, pooled and homogenized using a Miltenyi gentle MACS cell dissociator (Miltenyi Biotec, San Diego, California, United States). Infiltrated leukocytes were isolated by discontinuous Percoll gradient centrifugation and quantitated from the brain of WT and *db/db* mice as described previously [34,37]. Cells were counted and washed with 1 × fluorescence activated cell sorter buffer (0.5% BSA and 2 mM ethylenediaminetetraacetic acid). To identify for CD45^+^, CD11b^+^, CD3^+^, CD4^+^ and CD8^+^ cell populations, cells were stained using fluorescein isothiocyanate (FITC)-conjugated anti-CD45, phycoerythrin (PE) Cy7-conjugated anti-CD11b, PE-conjugated anti-CD3, PE Texas Red-conjugated anti-CD4 and allophycocyanin (APC)-conjugated anti-CD8 antibodies (eBiosciences, San Diego, California, United States) for 30 minutes at 4°C and then fixed with 4% PFA at 4°C for 15 minutes. Fluorescence minus one samples were prepared for detecting any spillover from other channel. Samples were analyzed by multi-color flow cytometry using FACS Aria and data were analyzed using FlowJo software (version 9.4.11) (TreeStar, Ashland, Oregon, United States) as described previously [34,37].

### Measurement of cytokines and chemokines

Brains were weighed and homogenized in a bullet blender (Next Advance, Averill Park, New York, United States) using glass beads as described previously [19,34]. The levels of cytokines and chemokines were measured in the brain homogenates by multiplex immunoassay using MILLIPLEX MAP Mouse Cytokine/Chemokine kit (Millipore, Billerica, Massachusetts, United States) as described previously [34].

### PCR array

The expression profile of multiple cytokines, chemokines and their receptors in the brains of mock- and WNV-infected WT and *db/db* mice was analyzed using a commercial RT^2^ Profiler inflammatory cytokines and receptors PCR Array (SABiosciences, Valencia, California, United States, Catalogue number: PAMM-011Z) as described previously [34]. cDNA from four animals from each group was pooled. The fold-change in infected brains as compared to mock was calculated after normalizing to the housekeeping genes.

### Statistical analysis

Unpaired student t-test using GraphPad Prism 5.0 (GraphPad Software, La Jolla, California, United States) was used to calculate *P* values of difference. For multiplex immunoassay, two-way analysis of variance (ANOVA) with the *post hoc* Bonferroni test was used to calculate *P* values. Differences of p < 0.05 were considered significant.

## Results

### Leukocyte infiltration in the brains of *db/db* mice after WNV infection

**Figure 1:**
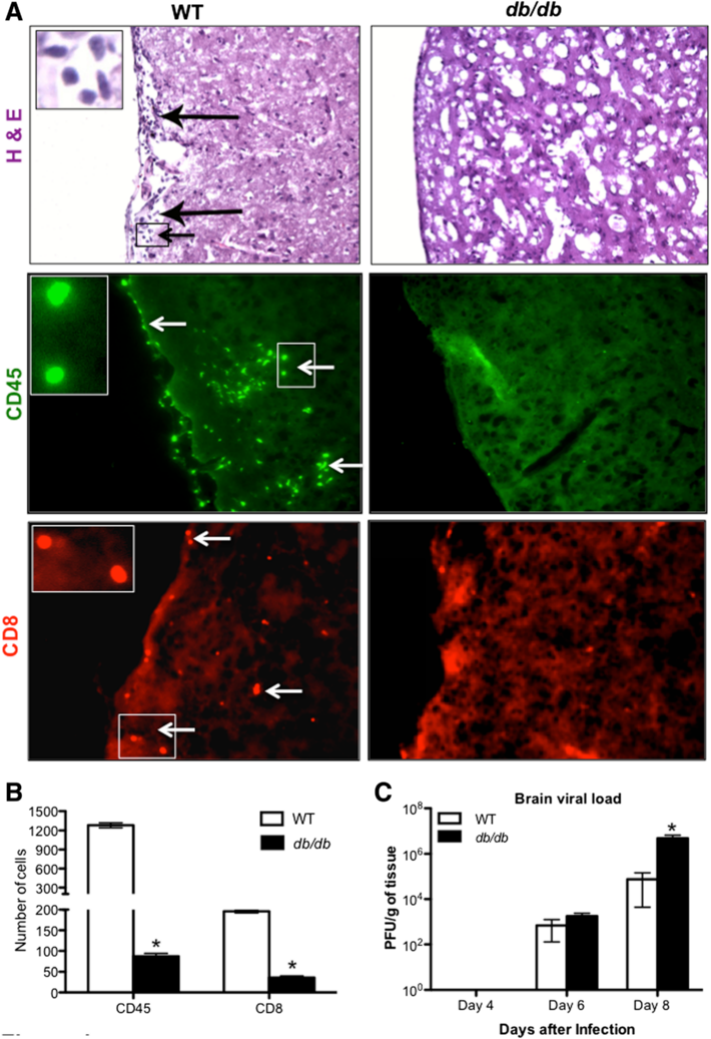
**Infiltration of leukocytes and viral load in the brains of WT and *db*/*db *mice after WNV infection. (A)** Cryopreserved brain sections from WNV-infected WT and *db/db* mice at day 8 after infection were stained with hematoxylin and eosin (H & E) and antibodies against CD45 (Green, white arrows and enlarged inset of the arrowed box) and CD8 (Red, white arrows and enlarged inset of the arrowed box). Black arrows and enlarged inset of the arrowed box on the H & E stained sections identify leukocytes infiltrating into the meninges of the WT brains. The photomicrographs demonstrate representative images obtained from two independent experiments (n = 4 per group). Bars, 20 μm. **(B)** Quantitative representation of total numbers of CD45 and CD8 positive cells from 15 different brain areas per section (total 2 brain sections per mice) from two independent experiments (n = 4 per group). *P <0.05. **(C)** Brain viral load was determined in WT and *db/db* mice, at days 4, 6, and 8 after infection by plaque assay using Vero cells and is reported as PFU per gram of tissue. Data represents the mean ± SEM, representing two independent experiments (n = 7-11 per group). *P <0.05. PFU, plaque-forming units; SEM, standard error of mean.

WT and *db/db* mice brain tissues were initially examined for histopathological changes following WNV infection. Hematoxylin and eosin (H & E) staining of brain sections from the WT mice demonstrated leukocyte infiltration along the meninges at day 8 after infection (Figure 1A). In contrast, fewer leukocytes were observed in the meninges of WNV-infected *db/db* mice. This observation was further confirmed by direct immunohistochemical analysis of the CD45 and CD8 antigen, which revealed markedly reduced staining of CD45 and CD8 positive cells in the *db/db* mice brains when compared with WT mice at day 8 after infection (Figure 1A). We further quantitated the CD45 and CD8 positive cells in the brain sections from four independent mice in each group. The average total cell number from 15 different brain areas per section (total 2 brain sections per mice) are depicted in Figure 1B. The number of CD45 and CD8 positive cells in *db/db* mice was significantly lower than in WT mice (*P* <0.05). We then measured WNV viral load in the brains of WT and *db/db* mice using a plaque assay. In contrast to leukocyte infiltration, significantly higher virus titer was observed in *db/db* mice brains when compared to the WT mice brains at day 8 after infection (Figure 1C, *P* >0.05).

**Figure 2:**
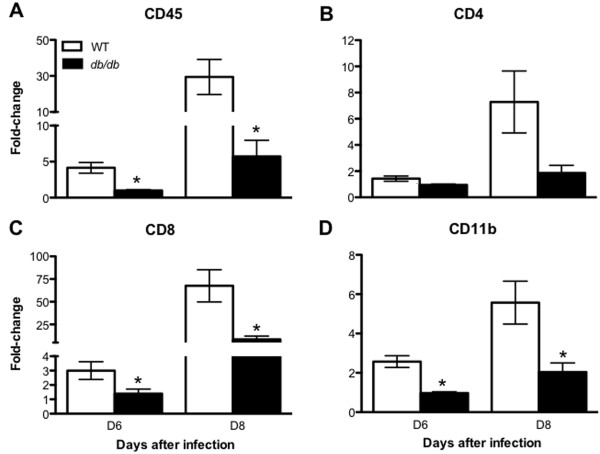
**WNV**-**induced leukocyte migration in the brain of WT and *db*/*db *mice.** qRT-PCR was conducted on RNA extracted from mock- and WNV-infected brains from WT and *db/db* mice at indicated time points to determine fold-change in **(A)***CD45*, **(B)***CD4*, **(C)***CD8* and **(D)***CD11b* gene expression. Changes in the levels of each gene were first normalized to the *β-actin* gene and then the fold-change in WNV-infected brain was calculated in comparison to corresponding mock-infected brain. Data represents the mean ± SEM, representing two independent experiments (n = 7 per group). *P <0.05. SEM, standard error of mean.

Furthermore, we compared the mRNA expression levels of *CD45*, *CD4*, *CD8*, and *CD11b* in the brains of WT and *db/db* mice at days 6 and 8 after infection using qRT-PCR. As expected, mRNA expression of *CD45*, *CD8*, and *CD11b* genes was up-regulated at day 6 after infection in WT mice, which further increased at day 8 after infection as compared to corresponding controls (Figure 2A, 2C, and 2D). However, this increase was attenuated in the brain of infected *db/db* mice. Expression levels of *CD45*, *CD8* and *CD11b* genes were significantly low in infected *db/db* mice when compared with infected WT mice at both days 6 and 8 after infection (*P* <0.05). No significant difference in the expression of *CD4* was observed between both groups (Figure 2B).

**Figure 3:**
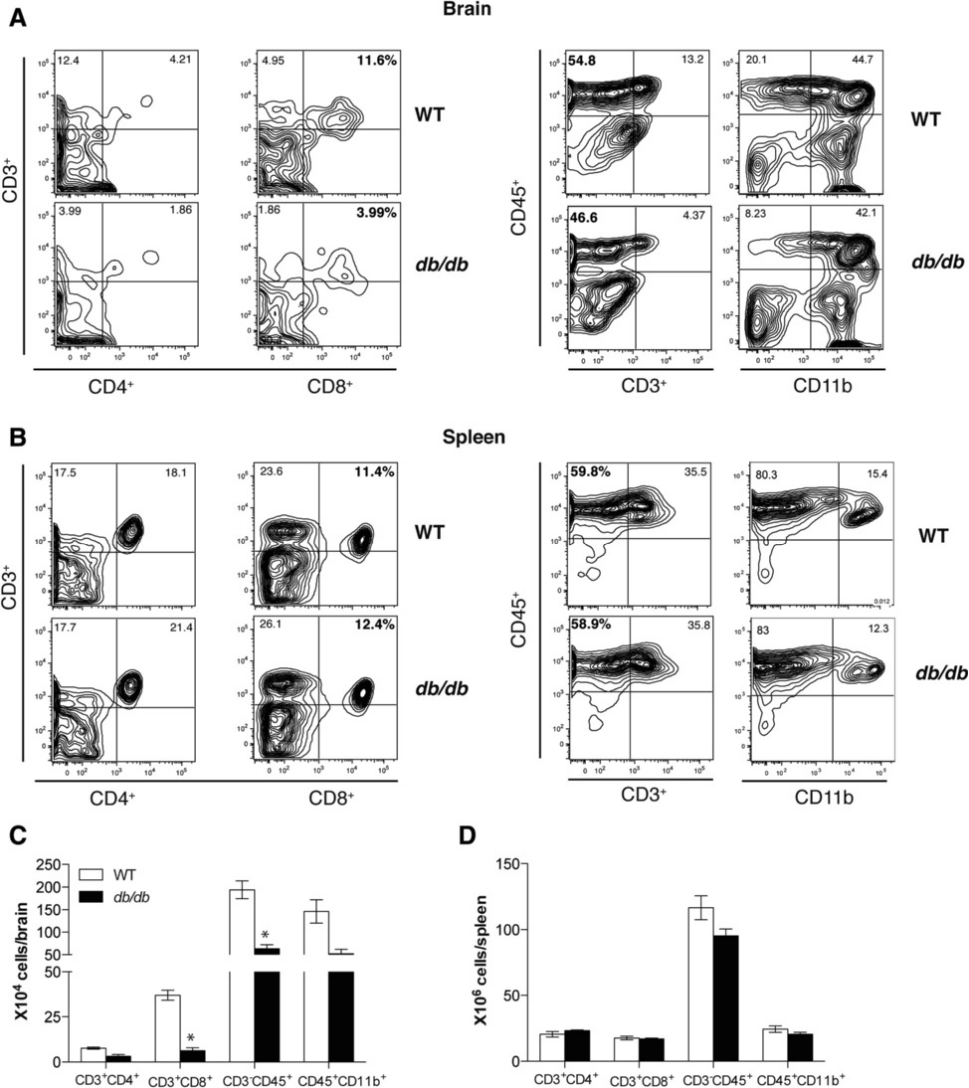
**Flow cytometric analysis of leukocyte infiltration in the brains and spleens of WNV-infected WT and *db*/*db *mice.** WT and *db/db* mice were infected with 10 PFU of WNV, and brains and spleens were harvested after extensive perfusion at day 8 after infection. Brain and spleen leukocytes were isolated and analyzed by flow cytometry. **(A and B)** Representative flow cytometry profiles demonstrating percentage of CD3^+^CD4^+^ T cells, CD3^+^CD8^+^ T cells, CD45^+^CD3^-^ leukocytes and CD45^+^CD11b^+^ cells in the brain and spleen at day 8 after infection in WT and *db/db* mice. Data represents two independent experiments (n = 6 per group). **(C and D)** Total number of CD3^+^CD4^+^ T cells, CD3^+^CD8^+^ T cells, CD45^+^CD3^-^ leukocytes and CD45^+^CD11b^+^ cells per brain and spleen were calculated. Data represents the mean ± SEM, representing two independent experiments (n = 6 per group). *P <0.05. PFU, plaque-forming units; SEM, standard error of mean.

To further verify these results we isolated leukocytes from the brain of WT and *db/db* mice at day 8 after infection and analyzed by flow cytometry. We stained the cells for CD45^+^, CD11b^+^, CD3^+^, CD4^+^ and CD8^+^ markers to analyze both T cells and non-T cell subpopulations. Consistent with the mRNA, histological analysis and immunohistochemistry results, there was a significant reduction in leukocyte infiltration in the brain of WNV-infected *db/db* mice when compared to WT mice. As shown in Figure 3A, the percentage of CD3^+^CD8^+^T cells in the brain of *db/db* mice was lower than in those of WT mice (3.9 versus 11.6%). Additionally, the percentage of CD45^+^CD3^-^ leukocytes was reduced in the *db/db* mice more so than in the WT mice (46.6 versus. 54.8%). Finally, the total number of CD3^+^CD8^+^ T and CD45^+^CD3^-^ cells trafficking into the brain of infected *db/db* mice was also significantly lower than in the infected WT mice (Figure 3C) (*P* <0.05). No statistically significant difference between the percentages or numbers of CD3^+^CD4^+^ T and CD45^+^CD11b^+^ cells in the brains of WT and *db/db* mice was identified.

Furthermore, we isolated leukocytes from the spleens of WT and *db/db* mice at day 8 after infection. Unlike the brain, equivalent percentage and numbers of CD3^+^CD4^+^ and CD3^+^CD8^+^ T cells, CD45^+^CD3^-^ cells as well as CD45^+^CD11b^+^ cells were observed in the spleens of WT and *db/db* mice (Figure 3B and 3D).

### Expression of chemokines and their receptors in the brains of *db/db* mice after WNV infection

**Figure 4:**
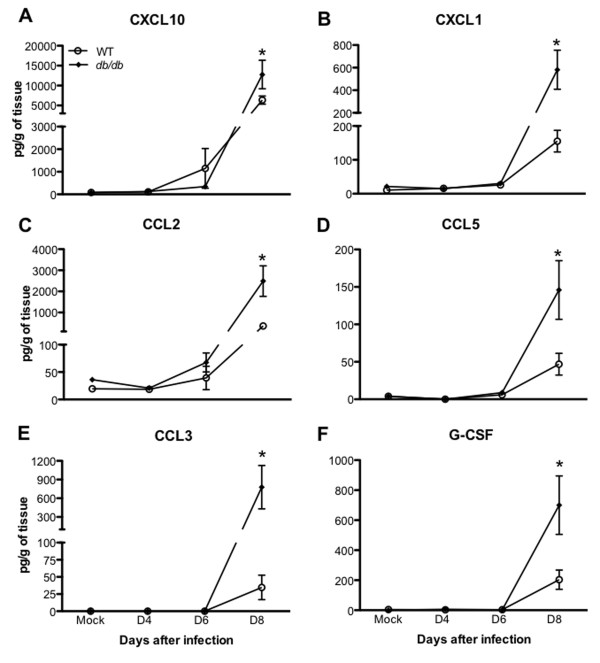
**Chemokines levels in the brains of WT and *db*/*db *mice after WNV infection.** Brains were harvested from WT and *db/db* mice at indicated time points and homogenized as described in the Materials and methods section. Levels of chemokines as noted in the figure **(A)** CXCL10, **(B)** CXCL1, **(C)** CCL2, **(D)** CCL5, **(E)** CCL3, **(F)** G-CSF were measured using multiplex immunoassay and are expressed as the mean concentration (pg/g of tissues) ± SEM, representing two independent experiments (n = 7 per group). *P <0.05. SEM, standard error of mean.

The migration of leukocytes into WNV-infected brain is associated with increased expression of several chemokines and their receptors in the brain [25]. It has been demonstrated that WNV-induced expression of chemokines such as CCL2, CXCL10 and chemokines receptors such as CCR5 and CCR2 promote the trafficking of leukocytes into the brain [25,29,39,40]. To determine whether decreased leukocyte accumulation in the brains of *db/db* mice could be indirectly attributed to the reduced expression of these chemokines in the brains of *db/db* mice, we analyzed the differential expression of various chemokines and their receptors in the brains of WT and *db/db* mice after WNV infection. mRNA levels of chemokines and their receptors were measured at day 8 after infection using a PCR array. WNV infection resulted in a dramatic increase in mRNA levels of key chemokines and their receptors in the brain of WT mice as compared to corresponding mock-infected mice (Table 2). In these mice, maximum increase of 113-, 279-, 394-, and 641-fold was observed in the levels of *CCL5*, *CCL2*, *CXCL9*, and *CXCL10* respectively. Moreover, there was almost 10-fold increase in the mRNA levels of these chemokines in the brain of infected *db/db* mice when compared to infected WT mice. The mRNA expression of chemokines receptors such as *CCR1*, *CCR5*, *CCR7* and *CXCR2* was also increased in the brains of infected *db/db* mice when compared to infected WT mice (Table 2). Similar to the mRNA expression, protein levels of the key chemokines such as CXCL10, CXCL1, CCL2, CCL5, CCL3, and G-CSF were increased in the brains of infected WT mice as measured by the multiplex immunoassay (Figure 4A-F). However, their levels were significantly elevated in the brains of *db/db* mice when compared to WT mice at day 8 after infection (*P* <0.05). Very low levels of these chemokines were detected in mock-infected mice. Moreover, there was no significant difference in the chemokine levels between mock-infected WT and *db/db* mice (Figure 4A-F).

**Table 2:** *Expression of chemokines and their receptors in the brain of WNV-**infected WT and *db****/****db *** **mice at day 8 after infection**

**Ligand**	**WT**	***db*/*db***	**Receptor**	**WT**	***db*/*db***
*CCL1*	-2.1	1.1	*CCR1*	6.1	10
*CCL2*	279	320	*CCR3*	5.0	4.4
*CCL3*	17	260	*CCR5*	2.2	5.0
*CCL4*	20	260	*CCR7*	3.8	8.1
*CCL5*	113	279	*CXCR2*	5.0	4.7
*CCL7*	86	243	*CXCR3*	-1.8	2.5
*CCL8*	21	43	*CXCR5*	-1.8	1.5
*CCL9*	6.6	3.5			
*CCL11*	-1.7	8.7			
*CCL12*	65	279			
*CXCL1*	4.0	24			
*CXCL5*	1.9	2.5			
*CXCL9*	394	3628			
*CXCL10*	641	4787			
*CXCL11*	7.6	80			
*CXCL13*	17	56			

*Changes in the levels of each gene was first normalized to the housekeeping genes and then the fold-change in WNV-infected brains was calculated in comparison to corresponding mock-infected brains.

### Expression of CAM in the brains of *db/db* mice after WNV infection

**Figure 5:**
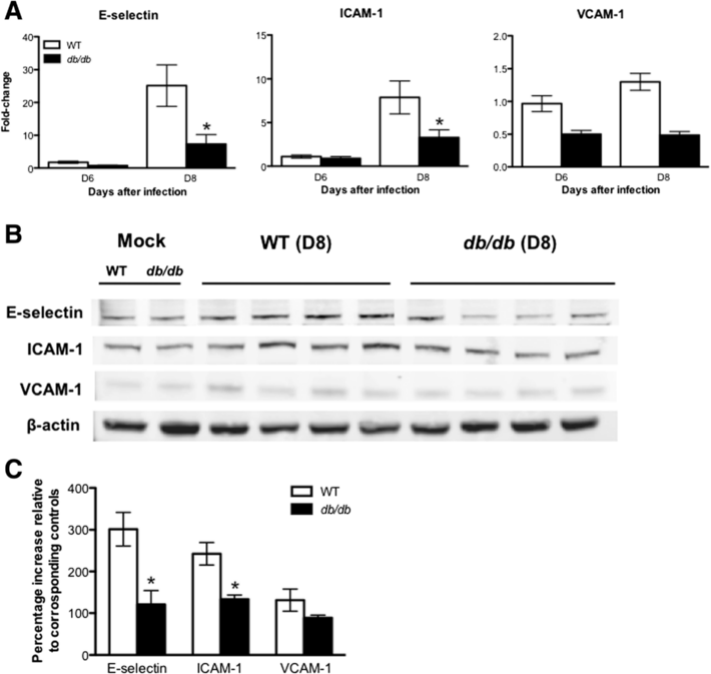
**WNV-induced CAM expression in the brains of WT and *db*/*db *mice. (A)** qRT-PCR was conducted on RNA extracted from mock- and WNV-infected brains from WT and *db/db* mice at indicated time points to determine the fold-change in *E-selectin*, *ICAM-1*, and *VCAM-1* gene expression. Changes in the levels of each gene were first normalized to the *β-actin* gene, and then the fold-change in WNV-infected brains was calculated in comparison to corresponding mock-infected brains. Data represents the mean ± SEM, representing two independent experiments (n = 7 per group). *P <0.05. **(B)** The protein levels of E-selectin, ICAM-1 and VCAM-1 were analyzed using Western blot analysis. Total brain lysates were separated by SDS-PAGE, transferred onto nitrocellulose membranes and immunoblotted with antibodies specific to E-selectin, ICAM-1, and VCAM-1. Equal loading was confirmed by re-probing with anti-β-actin antibody and the bands were detected using the Li-Cor Odyssey infrared method. **(C)** Quantitative analysis of Western blots results. Changes in the levels of each gene was first normalized to the β-actin gene and then the percentage increase in WNV-infected brains was calculated in comparison to corresponding mock-infected brains. Data represents the mean ± SEM, representing two independent experiments (n = 7 per group). *P <0.05. ICAM-1, intercellular cell adhesion molecule 1; VCAM-1, vascular cell adhesion molecule 1; SEM, standard error of mean.

**Figure 6:**
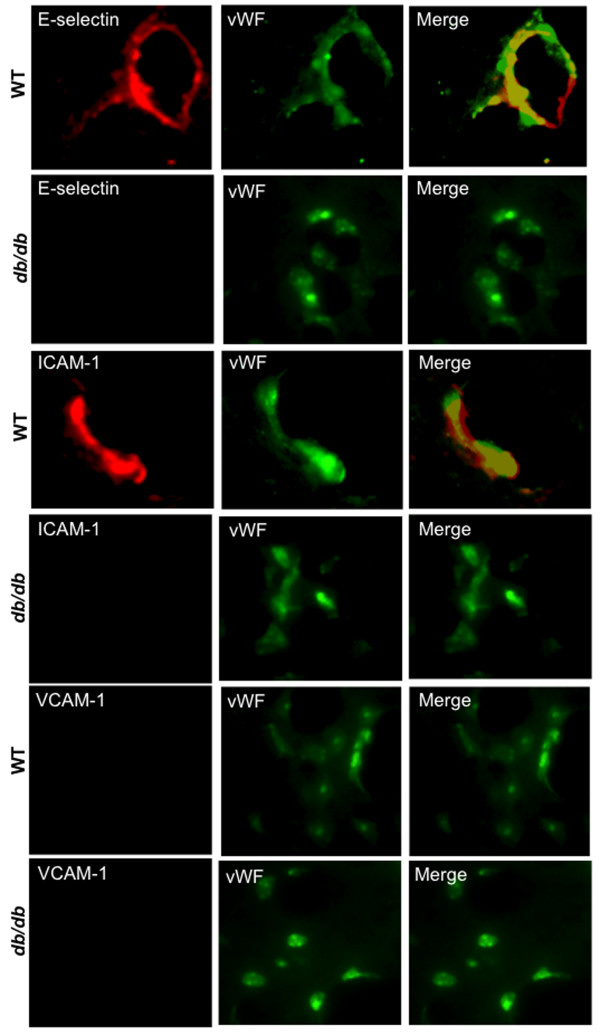
**Immunohistochemical analysis of CAM in the brains of WT and *db*/*db *mice after WNV infection.** Cryopreserved brain sections from WNV-infected WT and *db/db* mice at day 8 after infection were stained with antibodies against E-selectin, ICAM-1, VCAM-1 (Red), and vWF (Green). The photomicrographs demonstrate representative images obtained from two independent experiments (n = 4 per group). Bars, 20 μm. ICAM-1, intercellular cell adhesion molecule 1; VCAM-1, vascular cell adhesion molecule 1; vWF, von Willebrand factor.

WNV infection induces the expression of CAM such as ICAM-1, VCAM-1, and E-selectin, to facilitate leukocyte trafficking into the brain [31,36,41,42]. Since differences in leukocyte migration were observed, we analyzed the mRNA and protein expressions of E-selectin, ICAM-1, and VCAM-1 in the brain of WT and *db/db* mice after WNV infection. As anticipated, a 10- to 25-fold increase in the mRNA expression of *ICAM-1* and *E-selectin* were observed in the brains of WT mice compared to corresponding mock-infected mice at day 8 after infection (Figure 5A). In contrast, a modest three to seven-fold increase in the mRNA expression of *ICAM-1* and *E-selectin* were observed in the brains of infected *db/db* mice compared to corresponding mock-infected mice. The mRNA expression of both *E-selectin* and *ICAM-1* was significantly less in the infected *db/db* mice than in infected WT mice (*P* <0.05). However, there was no significant increase in the expression of *VCAM-1* in both WT and *db/db* mice after infection. Similar to mRNA expression, western blotting further demonstrated the increase in the protein expression of both E-selectin and ICAM-1 in the brains of WT mice at day 8 after infection. However this increase was markedly reduced in the brains of infected *db/db* mice (Figure 5B). As seen in Figure 5C, the increase in the expression of ICAM-1 and E-selectin was 250 to 300% in WT mice and 120 to 130% in *db/db* mice, compared with corresponding mock-infected mice. The protein levels of both E-selectin and ICAM-1 were significantly lower in the infected *db/db* mice when compared to infected WT mice (*P* <0.05). Moreover, immunohistochemical analysis revealed markedly reduced staining of E-selectin and ICAM-1 in the brains of *db/db* mice when compared with WT mice at day 8 after infection (Figure 6). Furthermore, increased expression of CAM in WT mice co-localized with endothelial cells (Figure 6).

Similar to the mRNA data, there was no significant increase in the protein expression of VCAM-1 in both WT and *db/db* mice after WNV infection (Figures 5 and 6).

### Expression of cytokines and their receptors in the brains of *db/db* mice after WNV infection

**Figure 7:**
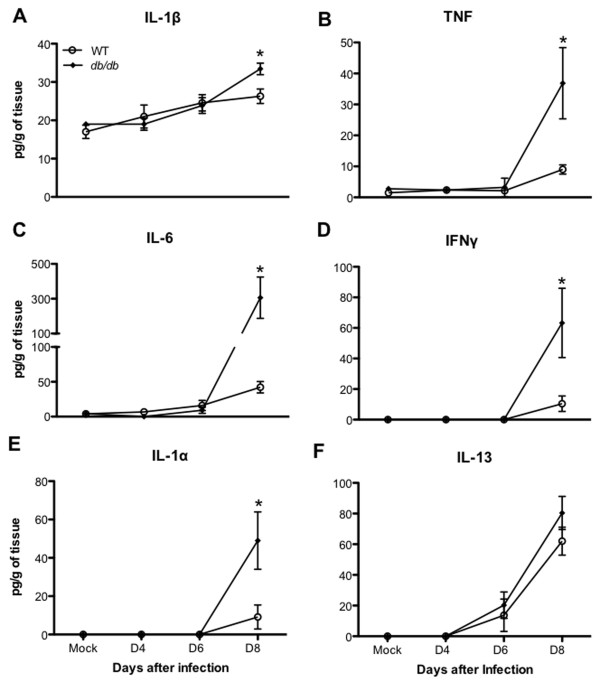
**Cytokines levels in the brains of WT and *db*/*db *mice after WNV infection.** Brains were harvested from WT and *db/db* mice at indicated time points and homogenized as described in the Materials and methods section. Levels of cytokines as noted in the figure **(A)** IL-1β, **(B)** TNF, **(C)** IL-6, **(D)** IFNγ, **(E)** IL-1α, and **(F)** IL-13 were measured using multiplex immunoassay and are expressed as the mean concentration (pg/g of tissues) ± SEM, representing two independent experiments (n = 7 per group). *P <0.05. SEM, standard error of mean.

WNV infection is associated with increased expression of several pro-inflammatory cytokines in the brain [25,43]. WNV-induced expression of pro-inflammatory cytokines such as IL-1β and TNF regulate leukocyte trafficking into the brain, and neuronal death after infection [32,38]. Diabetes is also associated with an enhanced inflammatory response to infections [6,7]. Consequently, we examined the expression levels of these key pro-inflammatory cytokines in the brains of WT and *db/db* mice after WNV infection. WNV infection resulted in a 3- to 10-fold increase in the mRNA expression of cytokines such as *IL-1β, TNF* and *IFNγ* in the brains of WT mice at day 8 after infection compared to corresponding mock-infected mice (Table 3). In contrast, there was an 11- to 113-fold increase in expression of the aforementioned cytokines in the brains of infected *db/db* mice compared to corresponding mock-infected mice. We also observed a 8-fold increase in the expression of *IL-1α* in the infected *db/db* mice. Increased expression of cytokines in the brain of WT and *db/db* mice was concomitantly associated with an increase in the expression of their corresponding receptors. The mRNA expression of *IL1R2* and *TNFRSF1B* was increased by 4- and 2-fold, respectively, in WT mice at day 8 after infection (Table 3). In *db/db* mice, similar to increased cytokine levels, there was a 4- to 9-fold up-regulation of mRNA expression of their receptors (Table 3). Similar to the mRNA expression, protein levels of IL-1β, TNF, IL-6, IFNγ, and IL-1α were also significantly elevated in the brains of *db/db* mice when compared to WT mice at day 8 after infection as measured by the multiplex immunoassay (Figure 7A-F) (*P* <0.05). However, IL-13 levels did not differ between WT and *db/db* mice (Figure 7F). Very low levels of these cytokines were detected in mock-infected mice, moreover, there was no significant difference in the cytokine levels between mock-infected WT and *db/db* mice (Figure 7A-F).

**Table 3:** *Expression of cytokines and their receptors in the brains of WNV-**infected WT and *db****/****db *** **mice at day 8 after infection**

**Ligand**	**WT**	***db*/*db***	***Receptor***	**WT**	***db*/*db***
*IFNγ*	10	113	*IL1R1*	1.5	1.5
*IL-1α*	1.8	8.1	*IL1R2*	4.4	9.4
*IL-1β*	3.3	11	*TNFRSF1A*	1.8	7.1
*TNF*	6.1	92	*TNFRSF1B*	2.3	4.1
*IL-4*	-2.1	-4.2			

*Changes in the levels of each gene was first normalized to the housekeeping genes, and then the fold-change in WNV-infected brains was calculated in comparison to the corresponding mock-infected brains.

### Activation of astrocytes in *db/db* mice after WNV infection

**Figure 8:**
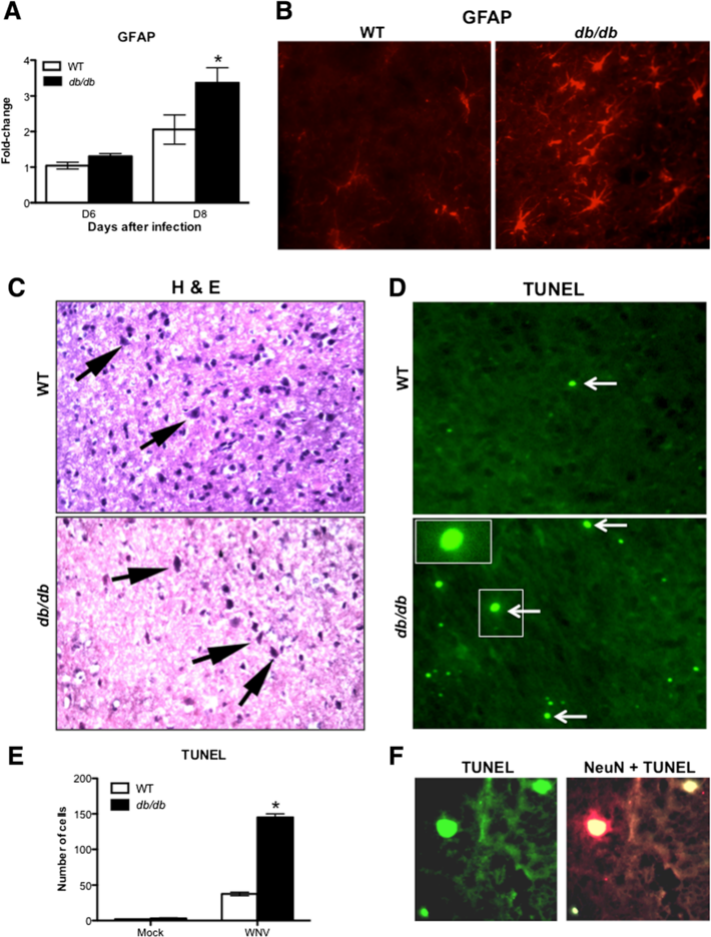
**Astrocyte activation and neuronal apoptosis in the brains of WT and *db****/****db *mice. (A)** qRT-PCR was conducted on RNA extracted from mock- and WNV-infected brains from WT and *db/db* mice at day 6 and 8 after infection to determine fold-change in *GFAP* gene expression. Change in the levels of *GFAP* gene was first normalized to the *β-actin* gene and then the fold-change in WNV-infected brains was calculated in comparison to the corresponding mock-infected brains. Data represents the mean ± SEM, representing two independent experiments (n = 7 per group). *p <0.05. **(B)** Cryopreserved brain sections from WNV-infected WT and *db/db* mice at day 8 after infection were stained for GFAP. Immunoreactivity of GFAP was higher in WNV-infected *db/db* mice. The photomicrographs demonstrate representative images obtained from two independent experiments (n = 4 per group). Bars, 20 μm. **(C)** Cryopreserved brain sections from WNV-infected WT and *db/db* mice at day 8 after infection were stained with H & E. Black arrows identify neurons. **(D)** TUNEL assay was conducted on cryopreserved brain sections from WNV-infected WT and *db/db* mice at day 8 after infection to evaluate neuronal apoptosis (Green, white arrows and enlarged inset of the arrowed box). The photomicrographs demonstrate representative images obtained from two independent experiments (n = 4 per group). Bars, 20 μm. **(E)** Quantitative representation of TUNEL-positive cells from 15 different brain areas per section (total 2 brain sections per mice) from two independent experiments (n = 4 per group). Number of TUNEL-positive cells were significantly higher in the brain of *db/db* mice. *P <0.05. **(F)** Co-immunostaining of TUNEL-positive cells with NeuN (neuronal cell marker). The photomicrographs demonstrate representative images obtained from two independent experiments (n = 4 per group). GFAP, glial fibrillary acidic protein; TUNEL, Terminal deoxynucleotidyl transferase dUTP nick end labeling; SEM, standard error of mean.

Astrocytes produce a wide variety of chemokines and cytokines upon exposure to pro-inflammatory stimuli. Since changes were observed in the chemokines and cytokines and taking into account the fact that activation of astrocytes is one of the major hallmarks of WNV infection [26], we next investigated the activation of astrocytes in WT and *db/db* mice at days 6 and 8 after WNV infection. The mRNA expression of *GFAP* was increased in WT mice at day 8 after infection compared to corresponding mock-infected mice. However, the increase in the *GFAP* expression was significantly higher in the infected *db/db* mice when compared to the infected WT mice (Figure 8A) (*P* <0.05). Similar to the mRNA expression, immunohistochemical analysis revealed increased GFAP expression in the WT mice at day 8 after infection. However GFAP immunoreactivity was more pronounced in the brain of *db/db* mice (Figure 8B).

### Neuronal death in *db/db* mice after WNV infection

Neuronal death is the hallmark of WNVE [44]. Therefore, we further evaluated the extent of neuronal death in the brains of WT and *db/db* mice after WNV infection. We examined H & E-stained sections of brain tissues from WT and *db/db* mice at day 8 after WNV infection. The neurons of *db/db* mice demonstrate eosinophilic cytoplasm and nuclear karyorrhexis, indicative of neuronal death, while neurons of WT mice from the same area of the brain are better preserved with basophilic cytoplasm and round nuclei (Figure 8C). WNV-induced neuronal apoptosis was further evaluated by direct TUNEL staining of WT and *db/db* brain tissues at day 8 after infection. TUNEL staining demonstrated markedly increased TUNEL-positive cells in the brain of *db/db* mice when compared to WT mice (Figure 8D). All the TUNEL-positive cells co-localized with NeuN (neuronal cell marker) (Figure 8F). Furthermore, we quantitated TUNEL-positive cells in the brain sections from four different mice in each group. The number of TUNEL-positive cells in the inflected *db/db* mice were significantly higher than in infected WT mice (Figure 8E) (*P* <0.05).

## Discussion

One of the hallmarks of WNVE is the accumulation of immune cells. The infiltration of immune cells is associated with increased production of neuroinflammatory molecules such as cytokines and chemokines and requires up regulation of CAM. In contrast, we demonstrate that WNVE in *db/db* mice is characterized by the reduced accumulation of leukocytes, despite increased production of pro-inflammatory cytokines and chemokines, and is associated with attenuated expression of CAM, increased brain viral load, activation of astrocytes, and enhanced neuronal death. These results, in conjunction with our previously published data, suggest that impaired migration of immune cells leads to reduced virus clearance from the brain, resulting in high mortality in WNV-infected *db/db* mice [19].

### Reduced leukocyte infiltration in the brains of *db/db* mice

WNVE is characterized by virus-associated pathological processes, including reaction of the brain resident cells and infiltration of inflammatory leukocytes, primarily monocytes and T cells, in the perivascular space and parenchyma, [25,29]. While infiltration of monocytes is protective, it also contributes to WNV-associated pathology [39,40,45]. T cells play an important role in protecting the host against WNV infection. While CD4^+^ T cells respond primarily in the periphery, CD8^+^ T cells migrate into the brain and clear WNV from infected neurons [43]. CD8^+^ T cells utilize perforin- and Fas ligand-dependent cytolytic mechanisms to limit WNV infection [46,47]. These cytolytic clearance mechanisms by effector CD8^+^ T cells limit viral burden and neurological disease, and outweigh the possible pathological effects of immune-targeted neuronal injury by CD8^+^ T cells. It has been demonstrated that mice lacking CD8^+^ T cells have increased viral burden in the brain and mortality rate when infected with WNV [30,46,47]. Similar to the previously published data, we demonstrate that brain WNV titer in *db/db* mice was significantly higher than in WT mice (Figure 1C) [19]. Although we also observed a delay in the induction of IFN-α response in the brains of *db/db* mice, IFN-α levels were similar in the brains of WT and *db/db* mice at day 8 after infection [19]. Moreover, as described above, adaptive immune responses (particularly T cell mediated immunity) is a predominant immune response and is essential for controlling WNV infection in the brain. In this study, we used a variety of experimental approaches to demonstrate that WNV-infected *db/db* mice had reduced levels of infiltrating CD45^+^ and CD8^+^ cells in the brain when compared to infected WT mice (Figures 1, 2, 3). Although several peripheral responses including antiviral immune responses (IFN-α, IgM, and IgG) and pro-inflammatory responses, were altered in the WT and *db/db* mice [19], we observed a similar number of immune cells including CD8^+^ T cells in the spleen of WNV-infected WT and *db/db* mice (Figure 3).

It is known that the diabetic condition reduces leukocyte adherence and transmigration [48,49]. Several *in vitro* studies have demonstrated significantly reduced transmigration of neutrophils and monocytes in response to various chemotactic stimuli across an *in vitro* transwell chamber or Boyden chamber in diabetic patients compared to non-diabetic controls [3,8,9,49,50]. Recently, an *in vivo* study using *db/db* mice revealed reduced infiltration of neutrophils in the brain after LPS administration [10]. Moreover, it has also been demonstrated that reduction in rolling, adhesion, and migration of leukocytes to the site of infection increased susceptibility of diabetic mice to polymicrobial sepsis [49]. Similarly, in our study, the presence of diabetes significantly alters leukocyte recruitment in the brain, resulting in a failure to clear WNV infection in the brains of *db/db* mice.

### Enhanced inflammation in the brains of *db/db* mice despite reduced leukocyte infiltration

WNV-induced expression of pro-inflammatory molecules such as CXCL10, CCL2 and TNF are essential for the trafficking of leukocytes into the brain [29,32,39]. Increased expression of cytokines and chemokines in a WNV-infected brain is usually associated with the enhanced trafficking of leukocytes into the brain [43]. We observed an increase in the various chemokine and cytokine levels in the brains of *db/db* mice despite reduced leukocyte infiltration (Figures 4 and 7, Tables 2 and 3). This effect may be due to the absence of CD8^+^ T cells which may lead to uncontrolled virus replication in resident brain cells, such as neurons and astrocytes, resulting in the increased production of cytokines and chemokines. It has been demonstrated that WNV-infected neurons directly contribute to the inflammation by secreting various chemokines and cytokines [29,38]. Moreover, the activation of astrocytes is one of the key pathogenic features of WNVE [26,51]. WNV-induced increased production of cytokines and chemokines has been demonstrated in the astrocytes [52-54]. In this study, we also demonstrate an increase in astrocytes activation in the brains of *db/db* mice when compared to WT mice (Figure 8A and B). These results suggest that WNV-infected neurons and activated astrocytes are the potential source of these inflammatory molecules in the brains of *db/db* mice. However, our data do not rule out the possibility of cytokine production by infiltrating NK cells and/or resident microglial cells in the brains of *db/db* mice. Moreover, fewer immune cells infiltrating into the brains of *db/db* mice may be hyperresponsive and producing increased amounts of pro-inflammatory mediators in the brains of these mice. Similarly, in our previous study, we also observed increased production of pro-inflammatory mediators in the serum of *db/db* mice when compared to WT mice [19]. These data collectively demonstrate that WNV infection in *db/db* mice led to the increased production of inflammatory mediators, both in the periphery and in the brain.

### Attenuated WNV-induced CAM expression in the brains of *db/db* mice

The other possible explanation for the reduced leukocyte migration observed in the brains of WNV-infected *db/db* mice is the expression of CAM. CAM are required for leukocyte migration into the brain [55]. WNV infection also increases the expression of CAMs such as ICAM-1, VCAM-1, and E-selectin, to facilitate leukocyte trafficking into the brain parenchyma [31,41,42]. It has been demonstrated that CAM are important for maintaining leukocytes in the brain following WNV infection. Moreover, leukocyte and macrophage infiltrates were decreased in the brains of ICAM-1^-/-^ mice after WNV infection [31]. Similarly, we observed an increased expression of ICAM-1 and E-selectin in the brains of WT mice after WNV infection, which correlates with leukocyte infiltration in these mice (Figures 5 and 6). Similar results have also been observed in other CNS-tropic viruses such as Theiler’s murine encephalomyelitis virus (TMEV) [56]. It has been demonstrated that TMEV infection induces the expression of CAM such as ICAM-1 and VCAM-1 in the mice brain, which plays an important role in mediating the infiltration of leukocytes into the brain [56-58].

In our study, significant up-regulation of CAM is observed at day 8 after infection in the brain of WT mice, which correlates with high virus replication (Figure 1C) [19]. In contrast, levels of both E-selectin and ICAM-1 were significantly lower in the brains of infected *db/db* mice despite a significantly high viral load in the brains of *db/db* mice when compared to WT mice (Figures 1C and 5). It has been demonstrated that patients with Type 2 diabetes exhibit an attenuated upregulation of ICAM-1, VCAM-1, and E-selectin after various stimuli such as LPS [3,11]. A recent study has demonstrated that the reduced expression of ICAM-1 plays a crucial role in decreased neutrophils recruitment into the brains of *db/db* mice after LPS-induced systemic inflammation [10]. Similarly, in WNV-infected *db/db* mice, reduced levels of CAM are insufficient to allow efficient leukocyte migration during WNVE despite high chemokines levels in the brain.

### Increased neuronal death in *db/db* mice

WNV-induced pro-inflammatory cytokines are known to modulate BBB permeability, activate glial cells, and mediate neuronal death, leading to the induction of lethal encephalitis [38,59,60]. In this study, we demonstrate significantly elevated levels of cytokines such as IL-1β, IL-6, TNF, IL-1α, and IFN-γ and their receptors in the brains of *db/db* mice at days 6 and 8 after WNV infection (Figure 7, Table 3), which correlated with increased levels of WNV in the brains of *db/db* mice [19]. IL-1β and TNF have been demonstrated to induce neuronal apoptosis after WNV infection [38]. Similarly, we also observed increased neuronal apoptosis in the brains of *db/db* mice when compared to WT mice (Figure 8C-F). Neuronal apoptosis is the hallmark of WNVE and an increase in neuronal apoptosis is associated with increased lethality following WNV infection [24,44]. Collectively, these results indicate a correlation between enhanced inflammatory response and increased neuronal apoptosis in the brains of *db/db* mice, thereby leading to increased mortality. These data are consistent with previous observations that *db/db* mice demonstrate a greater inflammatory response to various pathogens such as *Staphylococcus aureus*, *Porphyromonas gingivalis*, and *Trypanosoma cruzi*, in which a heightened inflammatory response was correlated with increased disease severity [7,61,62].

## Conclusions

Our previously published data demonstrated that the presence of diabetes attenuated peripheral antiviral immune responses leading to an increased WNV load in the serum, peripheral tissues, and brain [19]. In this study, we demonstrate that the presence of diabetes enhances WNV infection in the brain by inhibiting migration or accumulation of protective leukocytes in the brain, which correlates with reduced levels of CAM. Enhanced virus replication leads to increased levels of chemokines and cytokines in the brains of *db/db* mice, which mediates astrocytes activation and causes neuronal death, leading to lethal encephalitis and increased mortality in *db/db* mice. Collectively, these data and our previously published data [19] build the foundation for the development of a much needed therapeutic intervention to limit the progression of WNVE among diabetics.

## Abbreviations

BBB: Blood brain barrier; CAM: Cell adhesion molecules; CCL: Chemokine CC motif ligand; CCR: Chemokine CC motif receptor, CXCL, Chemokine CXC motif ligand; CXCR: Chemokine CXC motif receptor; ICAM-1: Intercellular cell adhesion molecule 1; IL: Interleukin; IL1R: Interleukin 1 receptor; IFN: Interferon; GFAP: Glial fibrillary acidic protein; LPS: Lipopolysaccharides; OCT: optimum cutting temperature; PBS: Phosphate buffered saline; PFA: Paraformaldehyde; PFU: Plaque forming units; TNF: tumor necrosis factor; TNFRSF1: Tumor necrosis factor receptor superfamily member 1; TUNEL: Terminal deoxynucleotidyl transferase dUTP nick end labeling; vWF: von Willebrand factor; WNV: West Nile virus; WNVE: WNV-associated encephalitis; WT: Wild-type.

## Competing interests

The authors declare that they have no competing interests.

## Authors’ contributions

MK and VRN designed and conducted experiments, analyzed results, and wrote the manuscript. KR and BO conducted experiments. SV and PVN were involved in early experimental design and discussions and provided intellectual input. KST examined and interpreted histopathology slides and provided intellectual input. All authors have read and approved the final version of the manuscript.
